# A Novel Machine-Learning Algorithm to Predict the Early Termination of Nutrition Support Team Follow-Up in Hospitalized Adults: A Retrospective Cohort Study

**DOI:** 10.3390/nu16152492

**Published:** 2024-07-31

**Authors:** Nadir Yalçın, Merve Kaşıkcı, Burcu Kelleci-Çakır, Karel Allegaert, Merve Güner-Oytun, Serdar Ceylan, Cafer Balcı, Kutay Demirkan, Meltem Halil, Osman Abbasoğlu

**Affiliations:** 1 Department of Clinical Pharmacy, Faculty of Pharmacy, Hacettepe University, 06100 Ankara, Türkiye; burcukelleci@hacettepe.edu.tr (B.K.-Ç.); kutay@hacettepe.edu.tr (K.D.); 2 Department of Biostatistics, Faculty of Medicine, Hacettepe University, 06100 Ankara, Türkiye; mervekasikci@outlook.com; 3 Department of Pharmaceutical and Pharmacological Sciences, KU Leuven, 3000 Leuven, Belgium; karel.allegaert@kuleuven.be; 4 Department of Development and Regeneration, KU Leuven, 3000 Leuven, Belgium; 5 Child and Youth Institute, KU Leuven, 3000 Leuven, Belgium; 6 Department of Hospital Pharmacy, Erasmus MC, 3015 GD Rotterdam, The Netherlands; 7 Department of Internal Medicine, Division of Geriatrics, Faculty of Medicine, Hacettepe University, 06100 Ankara, Türkiye; mguner54@gmail.com (M.G.-O.); serdar.ceylan@hacettepe.edu.tr (S.C.); cafer.balci@hacettepe.edu.tr (C.B.); meltemgulhan.halil@hacettepe.edu.tr (M.H.); 8 Clinical Nutrition Master’s Program, Hacettepe University, 06100 Ankara, Türkiye; osmana@hacettepe.edu.tr

**Keywords:** clinical nutrition, hospitalization, nutrition support team, medical nutrition therapy, oral intake, machine learning prediction

## Abstract

Background: For hospitalized adults, it is important to initiate the early reintroduction of oral food in accordance with nutrition support team guidelines. The aim of this study was to develop and validate a machine learning-based algorithm that predicts the early termination of medical nutritional therapy (the transition to oral feeding). Methods: This retrospective cohort study included consecutive adult patients admitted to the Hacettepe hospital (from 1 January 2018 to 31 December 2022). The outcome of the study was the prediction of an early transition to adequate oral feeding before discharge. The dataset was randomly (70/30) divided into training and test datasets. We used six ML algorithms with multiple features to construct prediction models. ML model performance was measured according to the accuracy, area under the receiver operating characteristic curve, and F1 score. We used the Boruta Method to determine the important features and interpret the selected features. Results: A total of 2298 adult inpatients who were followed by a nutrition support team for medical nutritional therapy were included. Patients received parenteral nutrition (1471/2298, 64.01%), enteral nutrition (717/2298, 31.2%), or supplemental parenteral nutrition (110/2298, 4.79%). The median (interquartile range) Nutritional Risk Screening (NRS-2002) score was 5 (1). Six prediction algorithms were used, and the artificial neural network and elastic net models achieved the greatest area under the ROC in all outcomes (AUC = 0.770). Ranked by z-value, the 10 most important features in predicting an early transition to oral feeding in the artificial neural network and elastic net algorithms were parenteral nutrition, surgical wards, surgical outcomes, enteral nutrition, age, supplemental parenteral nutrition, digestive system diseases, gastrointestinal complications, NRS-2002, and impaired consciousness. Conclusions: We developed machine learning models for the prediction of an early transition to oral feeding before discharge. Overall, there was no discernible superiority among the models. Nevertheless, the artificial neural network and elastic net methods provided the highest AUC values. Since the machine learning model is interpretable, it can enable clinicians to better comprehend the features underlying the outcomes. Our study could support personalized treatment and nutritional follow-up strategies in clinical decision making for the prediction of an early transition to oral feeding in hospitalized adult patients.

## 1. Introduction

According to the European Society for Clinical Nutrition and Metabolism (ESPEN) guidelines, optimal medical nutritional therapy (MNT) is one of the tasks of a nutrition support team (NST). Such teams consist of professionals such as physicians from different disciplines (geriatric medicine, intensive care units, pediatrics, surgery, gastroenterology, etc.), registered dietitian nutritionists (RDNs), nurses, and clinical pharmacists specially trained in enteral (EN) and parenteral (PN) nutritional therapy [1]. The initiation of MNT safely and with the ideal composition within a suitably chosen patient population is crucial to enhance the quality of overall patient care [2]. 

In a UK-based study, NST successfully converted inappropriate referrals for PN to EN in 41% of cases with a cost-saving effect [3]. NST was also associated with a reduction in the frequency of MNT complications such as catheter-related bloodstream infections (CRBSIs), electrolyte imbalances, and refeeding syndrome following the implementation of NST [4,5,6]. Concerning MNT, a cohort study illustrated the influence of an NST on the care of patients required or recommended for PN [3]. Following a well-organized training initiative for nurses overseen by the NST, the incidence of catheter-related sepsis in PN patients significantly declined from 71% before the NST to 29% in their initial year. Furthermore, 55 cases of PN (accounting for 41% of referrals) were prevented through judicious NST evaluations and the prompt initiation of enteral feeding. While the provision of nutrition, especially via enteral feeding, offers a consistent supply of essential nutrients, prolonged use may diminish the sensory enjoyment associated with traditional eating experiences and contribute to adverse gastrointestinal effects including diarrhea, constipation, compromised gut microbiome, and deficiencies in micronutrients. Therefore, reintroducing oral food consumption is important for eligible patients [7].

According to the ESPEN guideline on hospital nutrition, it is recommended to regularly evaluate, re-evaluate, and potentially customize the nutrition in each patient based on their specific needs. This assessment should occur at regular intervals, typically every three to five days, taking into account factors such as disease progression, monitoring of the ability to reintroduce oral food intake, and the patient’s status [8].

The nature of documented NSTs and the way they have been implemented vary, not only among different countries but also within a single country [9,10,11]. Therefore, there is a need for decision support systems to guide clinicians in order to prevent heterogeneity in clinical decision making and ensure standardization. It has been suggested that technological innovations used at the institutional level to facilitate timely referral to NST may improve the outcomes of changes in the organization of nutrition support for inpatients [12,13,14]. The initiation and termination of NST consultation and intervention in the right patient at the right time to prevent CRBSI, electrolyte imbalances, cachexia, and tube dependency by using clinical decision support systems has become essential for optimal MNT [15,16]. 

In this study, we aimed to develop and validate a machine learning (ML)-based algorithm that predicts the reason for f terminating MNT follow-up (the transition to oral feeding) by an NST using demographic and clinical parameters of adult patients admitted to the hospital.

## 2. Methods

### 2.1. Study Design and Population

The data of patients who were admitted to a tertiary care university hospital with a 1200-bed capacity in Turkey between 1 January 2018 and 31 December 2022 were retrospectively obtained from daily and standardized patient follow-up forms and electronic health record systems. Patients who were followed by an NST for at least 24 h and who did not die until the end of the follow-up period were included in the study. Patients aged 0–18 years who were followed up by the pediatric NST working independently from the adult NST were not included in this retrospective cohort study. The hospital’s NST includes general surgeons, geriatricians, intensive care unit (ICU) specialists, anesthesiologists, nurses, RDNs, and clinical pharmacists who have worked together for at least two years and have experience in clinical nutrition. As the first accredited NST in Turkey, quality control is carried out regularly [17]. The follow-up form encompasses essential patient information including age, gender, body mass index (BMI), reason for hospitalization, hospitalized ward, indication for nutritional therapy, Nutritional Risk Score (NRS)-2002, nutritional access route, duration of follow-up, reason for terminating follow-up, comorbidities, achievement of nutritional goals, and complications associated with nutritional therapy. These data were collected at the time of consultation with the NST.

Three different medical nutrition therapies were initiated by the NST in the adult patients included in the study: PN, EN, and supplemental PN. There were no patients on oral feeding at the time of referral. The wards where the patients were followed up were categorized into 3 different types to prevent heterogeneity in model performance: internal medicine wards, surgical wards, and ICUs. Similarly, the primary diagnosis (cancer, diseases of the digestive system, and others) and the reason for the initiation of MNT (gastrointestinal system (GIS) complications, surgical outcomes, impaired consciousness, and others) were also categorized. The dataset comprised 2298 adults, and a comprehensive set of variables pertaining to these adults was obtained during the data-gathering process. Several variables (such as the length of stay, energy, protein, and achievement of nutritional therapy) have missing values. These variables were excluded from the analysis due to a high percentage of missing data. Using demographic (age, gender, and body mass index) and clinical parameters (admitted ward, diagnosis, comorbidities, NRS-2002, type of nutrition, and reason for MNT) as input, artificial intelligence (AI) models (Shapley additive explanations, recursive feature elimination, and Boruta methods) were designed to predict why the patients were terminated from NST follow-up (the transition to oral intake vs. other reasons), and the highest-performing model was determined.

### 2.2. Statistical Analysis

Analyses were conducted using the free and open-source R software (version 4.4.1, http://www.rproject.org (accessed on 5 July 2024)). The dataset was randomly divided into a training set (70%) and a testing set (30%). Using the training set, feature selection was performed to determine the important variables in the dataset. Boruta, Shapley additive explanations (SHAP), and recursive feature elimination (RFE) are commonly used methods for feature selection and were applied separately here. Unlike SHAP and RFE methods, Boruta clearly defines whether a feature is important or unimportant and determines the optimal number of features [18,19]. Therefore, of these three methods, we decided to use Boruta. Boruta is a random forest-based feature selection method that generates random versions of the real features in the dataset, called shadow features. The maximum importance value among these shadow features is determined as the cut-off value. Features with importance values above this cut-off value are determined as important and features with importance values below this cut-off value are determined as unimportant. The process is repeated many times to ensure that the importance of features is not determined by chance. 

After determining the important features, classification models were developed using different algorithms. These algorithms were artificial neural networks (ANNs), elastic-net regularized generalized linear models (ENs), random forest (RF), extreme gradient boosting (XGBoost), a support vector machine with radial basis kernel function (SVM-radial), and a support vector machine with linear kernel function (SVM-linear). Subsequent to the configuration of input variables, six distinct ML methods were employed for the classification task. Five-fold cross-validation was implemented on the training set during the model development process to improve the validity of the classification models. Given that the five-fold cross-validation method involves the partitioning of the training data into distinct training and validation sets, a discrete validation set was not employed during the dataset division process. For smaller datasets (n < 10,000), five-fold cross-validation is often sufficient because it requires less computational power [20]. To mitigate the risk of overfitting inherent in machine learning methods, parameter optimization was undertaken using the tuneLength parameter within the caret package, facilitating the automated adjustment of model parameters [21]. The following parameters were automatically optimized using the tuneLength argument: the number of hidden units and weight decay in the ANN algorithm; alpha and lambda parameters in the EN algorithm; the number of random variables in each tree in the RF algorithm; the number of boosting iterations, the maximum depth of the decision tree, the learning rate, the subsample ratio, and minimum loss reduction in the XGBoost algorithm; the cost parameter in the SVM-linear algorithm; and the cost and sigma parameters in the SVM-radial algorithm.

**Figure 1:**
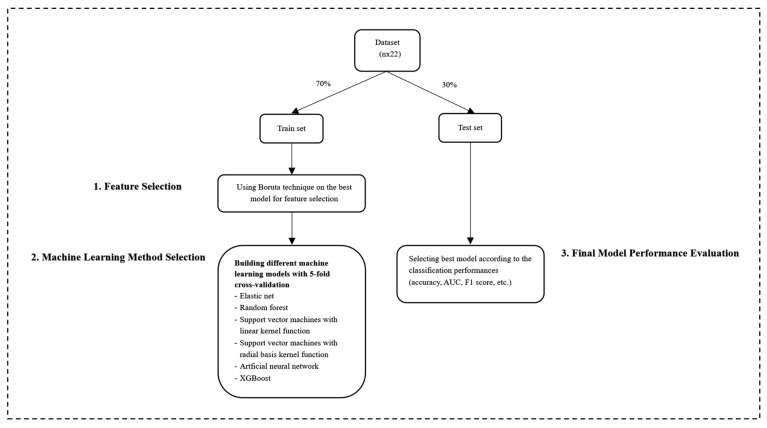
Flow chart of ML procedure.

Test set performances of the models were evaluated based on measures such as accuracy, sensitivity, specificity, F1 score, and AUC. Complete-case analysis was performed on the ML models. The Boruta [19] package was used for variable selection and the caret [21] package was used to obtain classification models and parameter optimization. The GMDH2 [22] and pROC [23] packages were used to calculate the performance measures (Table 1). The seed number was set to 1234 for reproducibility. The codes are available at https://github.com/mervekasikci/NST (accessed on 5 July 2024). As there were no missing values for patients in the selected variables, no efforts were made to perform any data imputation. The steps for classification are summarized in the flow chart (Figure 1). The Transparent Reporting of a multivariable prediction model for Individual Prognosis or Diagnosis (TRIPOD) checklist was used to enhance the dependability and significance of clinical prediction models by advocating for clear and precise reporting [24] (Supplementary Table S1).

### 2.3. Ethics Approval

This retrospective study design received ethics approval from the local ethics committee (decision no: 2019/08-02, date: 19 March 2019). All procedures performed in studies involving human participants were in accordance with the ethical standards of the institutional and/or national research committee and with the 1964 Helsinki Declaration and its later amendments or comparable ethical standards. The need for consent to participate was waived by the Hacettepe University Institutional Review Board due to the retrospective study design.

## 3. Results 

### 3.1. Baseline Characteristics

A total of 2298 (55.92% male) adult inpatients who were followed by an NST for MNT were included in the study. Of those, 990 (43.08%) patients were transferred to oral feeding at the termination of the NST follow-up. The mean (SD) age of the patients was 61.52 (17.20). There were no patients whose follow-up was not terminated by the NST during the study period. The mean (SD) BMI of the patients was 23.70 (5.35) kg/m^2^. The median (IQR) of NRS-2002 was 5 (1). 

The most common ward to which patients were admitted was internal medicine (40.17%) and the most common diagnosis was cancer (64.58%). The most common type of MNT initiated by the NST was PN, while the most common reason for MNT was the inability to use the gastrointestinal system (69.06%). The most common comorbid disease in hospitalized patients was diabetes mellitus (20.37%) (Table 2). 

### 3.2. Feature Selection

**Figure 2:**
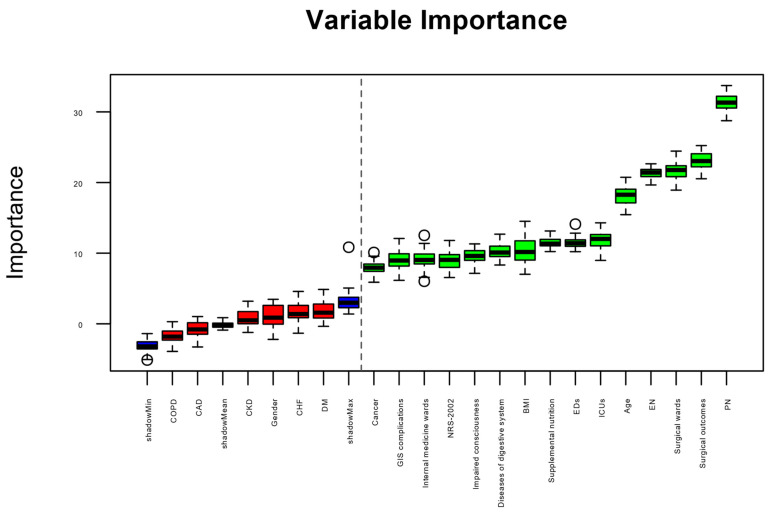
Feature importance according to the Boruta method. Blue boxes represent shadow attributes, green color signifies significant attributes, and red boxes denote attributes considered unimportant. CAD: coronary artery disease, COPD: chronic obstructive pulmonary disease, CKD: chronic kidney disease, DM: diabetes mellitus, CHF: congestive heart failure, ICUs: intensive care units, BMI: body mass index, NRS-2002: Nutrition Risk Screening Score-2002, GIS: gastrointestinal system, EN: enteral nutrition, PN: parenteral nutrition. The dashed vertical line represents the discrimination between variables that were and were not important according to Boruta algorithm.

The outcomes of feature screening utilizing the Boruta algorithm are depicted in Figure 2. The 10 features most strongly associated with the reason for terminating MNT follow-up (transition to oral feeding vs. others), ranked by z-value, were PN, surgical wards, surgical outcomes, EN, age, supplemental PN, diseases of digestive system, GIS complications, NRS-2002, and impaired consciousness.

### 3.3. Comparison of Model Performance

**Figure 3:**
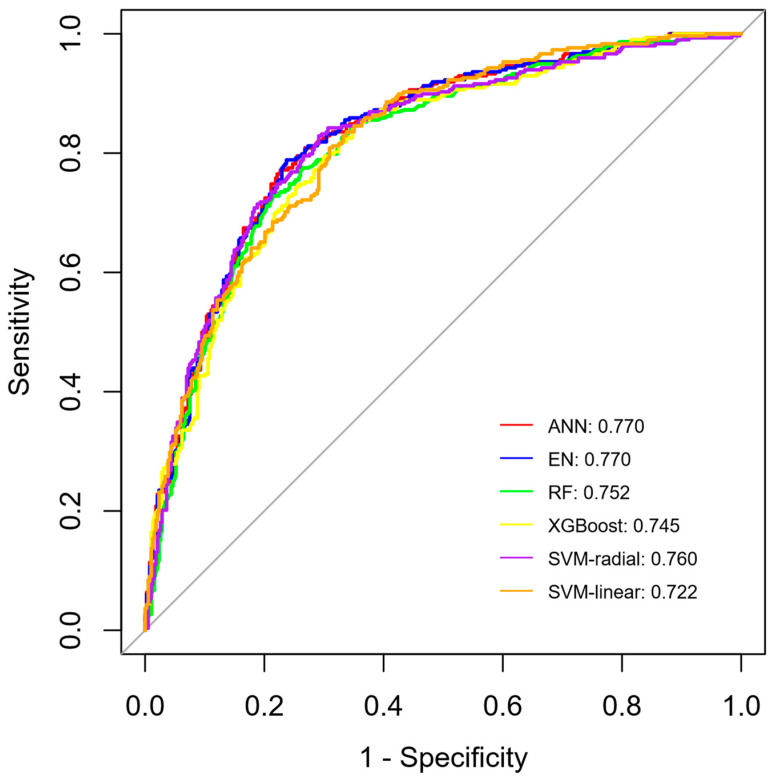
ROC curves from six models. ANN: artificial neural network, EN: elastic net, RF: random forest, XGBoost: extreme gradient boosting, SVM: support vector machine.

We generated six ML models to predict the early termination of follow-up. Figure 3 illustrates the discriminant performance of the six models concerning ROC curves. These ROC curves show that among the six models, the ANN and EN models (AUC = 0.770) had the best predictive effect on the reason for terminating MNT follow-up, followed by SVM-radial (AUC = 0.760), RF (AUC = 0.752), XGBoost (AUC = 0.745), and SVM-linear (AUC = 0.722). In line with the AUC values of ANN and EN models, 77% of patients were correctly classified as patients whose NST follow-up was terminated due to oral intake or other reasons. In comparison to the ANN and EN models, which exhibited an AUC of 0.770, the predictive performance of SVM-radial, RF, XGBoost, and SVM-linear was found to be suboptimal in the context of predicting the reason for terminating MNT follow-up by the NST in adult patients. While the AUC values of the models do not show a significant difference, ANN and EN methods provide the highest AUC values.

A comprehensive array of performance measurements for these six models is presented in Table 3. The ANN model has the highest sensitivity, NPV, detection rate, and detection prevalence with values of 0.772 and 0.814, respectively. On the other hand, the EN model has higher specificity and PPV, with values of 0.771 and 0.720, respectively. The other performance measures have the highest values for ANN and EN, equally for both models (Table 3).

## 4. Discussion

The generated ML model used to predict the early termination of MNT at follow-up has high accuracy and increases the trustworthiness of the model’s predictive ability. The demographic (age and gender) and clinical parameters (BMI, NRS-2002, ward, diagnosis, type of MNT, indication for MNT, and comorbidities) of the patients were categorized, determined, and introduced to the ML algorithms as input, and the model aimed to predict whether the successful transition to oral intake was achieved as output. Not only does an adequate MNT play an important role in patients’ health-related outcomes but predicting the ability of oral intake after MNT is also valuable in order to prove the positive effect of MNT interventions. This study is unique in its demonstration of the early prediction of the success of MNT and the decision-making process in terms of terminating follow-up before actual MNT intervention. Although it is known that the incidence of EN (0.59%) administration is higher than PN (0.46%) in the total population [25], the most important reason for the opposite result in our study is that our hospital is a tertiary referral care university hospital hosting complicated patients. 

Despite the widespread adoption of digital technologies and AI in medicine, their utilization in the field of nutrition remains relatively uncommon [26]. Many studies have been performed to show a relationship between assessment tools and balancing diet or losing weight [26,27,28,29]. Machine learning is emerging as a novel tool in clinical nutrition. Through a comprehensive ML approach, it becomes possible to achieve extensive screening [29,30]. Since it has been underlined that it is essential to identify and address the nutritional needs of medically complex inpatients [30], estimating patient status after MNT interventions has an important value.

In recent years, clinical audits have been employed to evaluate and enhance the quality of care across various healthcare domains including MNT. The parameters used to audit the quality of nutritional care concentrate on ten aspects pertinent to food and nutritional care, encompassing the availability, provision, and presentation of food, information about the provided food, the dining environment, nutritional screening and assessment, care planning, assistance with meals, monitoring of intake, general indicators, and health promotion [31]. A proficient NST plays a crucial role in overseeing numerous parameters essential for auditing the quality of nutritional care. An NST is responsible for assessing and, in collaboration with primary healthcare teams, overseeing MNT for patients who require or are likely to require it. The provision of nutrition care and the administration of nutrition support therapy should follow a structured series of steps incorporating feedback loops. These steps encompass nutrition screening, formal nutrition assessment, the development of a nutrition care plan, execution of the plan, patient monitoring, evaluation of the plan, assessment of the care setting, and, if necessary, the adjustment of the plan or the termination of therapy [32]. Consistent with previous statements, in the present study, an experienced NST monitored every aspect of patients’ MNT from the screening of patients to the termination of therapy in order to provide high-quality nutritional care.

Older adult patients, due to an increased nutritional risk rate and functional degeneration, frequently present with multiple conditions, rendering them more susceptible to both disease and malnutrition. Consequently, these individuals become the focus of targeted nutrition support [33]. Aging has an impact on the success of MNT, the incidence of complications, and other health-related outcomes. On the other hand, although a direct causal relationship cannot be established, there exists a significant correlation between inadequate oral health and malnutrition in elderly patients [34]. In this study, age was shown to be one of the most important variables in the success of nutrition therapy (the termination of nutrition therapy and (re)gaining adequate oral intake).

Using ML in a propensity-matched analysis, it was found that the initiation of early oral and/or EN within the first 3 days of hospital admission was associated with increased rates of discharge [35]. The timing and characteristics of the MNT are also very important for surgical patients since malnutrition is associated with worse outcomes [36]. Perioperative nutritional follow-up conducted by an experienced NST is imperative for these patients. This follow-up should persist until the patient is capable of resuming oral intake of sufficient food [36]. The existing ESPEN guidelines articulate that postoperative oral nutritional intake should be sustained without interruption, and the initiation of oral intake, including clear liquids, is recommended within hours following surgery for the majority of patients [37]. Perioperative MNT is warranted for individuals with pre-existing malnutrition or those identified as being at nutritional risk, when it is anticipated that a patient will be unable to consume food for a period exceeding five days in the perioperative phase, or patients expected to experience low oral intake and who are unable to maintain a dietary intake exceeding 50% of the recommended levels for a duration surpassing seven days [37]. In line with the recommendations for this study as per the model, besides age, EN and PN, the surgical ward, and outcomes emerge as the most crucial variables for discerning a patient’s adequate oral intake after MNT interventions by the NST.

The integration of AI technologies holds the potential to expedite the pursuit of optimal health and wellbeing by providing precise, personalized dietary recommendations and fostering the development of predictive and preventive guidelines for enhanced health promotion and disease management. Within the framework of national healthcare systems, AI stands to assist physicians in the selection of appropriate therapies, timely dose adjustments, and the identification of patients requiring more comprehensive or urgent examinations, distinguishing them from those with well-managed metabolic conditions. Moreover, AI has the capacity to alleviate the burden on healthcare professionals by minimizing the time devoted to in-person consultations, thereby reducing waiting times at medical facilities and contributing to an overall reduction in healthcare expenditures [26].

The severity of acute illness or disease can be equally influential in contributing to nutritional risk when compared to suboptimal nutritional status. The contemporary healthcare landscape underscores the importance of associating assessment and treatment with quantifiable indicators of success. The prospective implementation of AI systems holds the promise of facilitating the realization of effective nutritional interventions in the future [28].

Regarding the techniques used, random forest exhibits greater robustness in scenarios characterized by limited data as it manifests reduced susceptibility to overfitting [27]. For this reason, in this study, the random forest-based Boruta method was used as a feature selection method.

Despite achieving a targeted sample size least 20 times the number of dependent variables (input) and adopting a retrospective methodology to capture real-life data within the study population, it is imperative to acknowledge certain limitations inherent in this study. The utilization of data exclusively from a single center imposes constraints on the heterogeneity of the dataset and its applicability elsewhere. In order to enhance the generalizability of the models, it is necessary to conduct multicenter studies that include a larger representation of patients. The other limitations of the study are that other important variables such as the length of stay, energy, protein, and the achievement of nutritional therapy targets could not be included in the study because of a large number of missing data. Holdout (splitting the dataset into training and testing sets) and cross-validation methods were performed to decrease the possible overfitting of models. However, the risk that the simplified use of cross-validation may still lead to overfitting is another limitation of the study. Moreover, caution is warranted regarding the generalizability of the findings to diverse populations and other healthcare facilities, and the researchers duly recognize the imperative for additional validation to bolster the robustness of the study outcomes. The validation of the presented models should be broadened by conducting various research studies including diverse clinical settings and patient groups.

## 5. Conclusions

To the best of our knowledge, this is the first study to use an ML-based model to predict an early transition to oral intake with the Boruta method by means of important input parameters such as PN initiation, surgical ward admission, EN initiation, and age. The ML-based model developed and validated within this study for the early prediction of the transition to oral feeding is anticipated to serve as a directive tool in optimizing the management of NST responsibilities. This is expected to enhance the efficacy of the NST workload, bolster the confidence of NST staff, achieve nutritional targets immediately, and mitigate the risk of compromising the nutritional status of patients due to the early discontinuation of MNT. In an effort to expand the availability of the developed ML models to a broader range of healthcare professionals and facilitate their use, the development of mobile applications and web tools is being considered by providing detailed guidelines and training to health professionals in future work.

## Figures and Tables

**Table 1:** Explanations on performance measures used for classification models.

Measure	Formula	Definition
Accuracy	$\frac{tp+tn}{tp+fn+fp+tn}$	Ratio of correctly classified samples to the total number of samples.
Sensitivity (Recall)	$\frac{tp}{tp+fn}$	Ability of a model to correctly identify positive samples
Specificity	$\frac{tn}{tn+fp}$	Ability of a model to correctly identify negative samples
Positive Predictive Value (Precision—PPV)	$\frac{tp}{tp+fp}$	Indication of how many of the samples classified as positive by the model are actually positive
Negative Predictive Value (NPV)	$\frac{tn}{tn+fn}$	Indication of how many of the samples classified as negative by the model are actually negative
Balanced Accuracy	$\frac{Sensitivity+Specificity}{2}$	Arithmetic means of sensitivity and specificity
F1 Score	$\frac{2\times Precision\times Recall}{Precision+Recall}$	Harmonic mean of precision and recall
Area Under ROC Curve (AUC)	$\frac{1}{2}\sum_{i=1}^{n}\left({fp}_{i+1}-{fp}_{i}\right)({tp}_{i+1}-{tp}_{i})$	Indication of how well the classes is separated from each other according to the model obtained

*tp*: true positive, *tn*: true negative, *fp*: false positive, *fn*: false negative.

**Table 2:** Descriptive statistics of the study group.

		Reason for Follow-Up	
	Total (n = 2298)	Oral (n = 990)	Others (n = 1308)
Age, mean (SD)	61.52 (17.20)	57.91 (15.97)	64.26 (17.59)
BMI, mean (SD)	23.70 (5.35)	24.39 (5.39)	23.17 (5.27)
NRS-2002, median (IQR)	5 (1)	4 (1)	5 (1)
Gender, n (%)			
*Male*	1285 (55.92%)	562 (43.74%)	723 (56.26%)
*Female*	1013 (44.08%)	428 (42.25%)	585 (57.75%)
Wards, n (%)			
*Internal medicine wards*	923 (40.17%)	328 (35.54%)	595 (64.46%)
*Surgical wards*	720 (31.33%)	483 (67.08%)	237 (32.92%)
*ICUs*	655 (28.5%)	179 (27.33%)	476 (72.67%)
Diagnosis, n (%)			
*Cancer*	1484 (64.58%)	741 (49.93%)	743 (50.07%)
*Diseases of digestive system*	228 (9.92%)	145 (63.6%)	83 (36.4%)
*Other*	586 (25.5%)	104 (17.75%)	482 (82.25%)
Type of MNT, n (%)			
*PN*	1471 (64.01%)	880 (59.82%)	591 (40.18%)
*EN*	717 (31.2%)	89 (12.41%)	628 (87.59%)
*Supplemental PN*	110 (4.79%)	21 (19.09%)	89 (80.91%)
Indication for MNT, n (%)			
*Inability to use GIS*	1578 (69.06%)	610 (38.66%)	968 (61.34%)
*Surgical outcomes*	342 (14.97%)	259 (75.73%)	83 (24.27%)
*Impaired consciousness*	154 (6.74)	16 (10.39%)	138 (89.61%)
*Other*	211 (9.23)	101 (47.87%)	110 (52.13%)
Comorbidities, n (%)			
*DM*	468 (20.37%)	172 (36.75%)	296 (63.25%)
*CAD*	296 (12.88%)	101 (34.12%)	195 (65.88%)
*COPD*	149 (6.48%)	53 (35.57%)	96 (64.43%)
*CKD*	127 (5.50%)	36 (28.35%)	91 (71.65%)
*CHF*	117 (5.09%)	26 (22.22%)	91 (77.78%)

BMI: Body Mass Index (kg/m^2^), NRS-2002: Nutrition Risk Screening Score-2002, IQR: interquartile range, ICU: intensive care unit, PN: parenteral nutrition, EN: enteral nutrition, MNT: medical nutrition therapy, GIS: gastrointestinal system, DM: diabetes mellitus, COPD: chronic obstructive pulmonary disease, CKD: chronic kidney disease, CAD: coronary artery disease, CHF: congestive heart failure.

**Table 3:** Performance measures of classification models.

	ANN	EN	RF	XGBoost	SVM-Radial	SVM-Linear
Accuracy	**0.770**	**0.770**	0.754	0.745	0.762	0.722
Sensitivity	**0.772**	**0.768**	0.738	0.742	0.742	0.721
Specificity	**0.768**	**0.771**	0.765	0.747	0.778	0.722
PPV	**0.719**	**0.720**	0.707	0.693	0.720	0.666
NPV	**0.814**	**0.813**	0.792	0.790	0.797	0.771
Baccuracy	**0.770**	**0.770**	0.752	0.745	0.760	0.722
F_1_ Score	**0.744**	**0.744**	0.722	0.716	0.731	0.692
AUC (95% C.I.)	**0.770** **(0.738–0.802)**	**0.770** **(0.738–0.801)**	0.752 (0.719–0.785)	0.745 (0.712–0.778)	0.760 (0.728–0.792)	0.722 (0.688–0.755)

ANN: artificial neural network, EN: elastic net, RF: random forest, XGBoost: extreme gradient boosting, SVM: support vector machine, PPV: positive predictive value, NPV: negative predictive value, Baccuarcy: balanced accuracy, AUC: area under the ROC curve, C.I.: confidence interval.

## Data Availability

The original contributions presented in the study are included in the article, further inquiries can be directed to the corresponding author.

## Supplementary Materials

The following supporting information can be downloaded at: https://www.mdpi.com/article/10.3390/nu16152492/s1, Table S1. TRIPOD Checklist: Prediction Model Development and Validation.
